# A novel akermanite/poly (lactic-co-glycolic acid) porous composite scaffold fabricated via a solvent casting-particulate leaching method improved by solvent self-proliferating process

**DOI:** 10.1093/rb/rbx014

**Published:** 2017-06-20

**Authors:** Yao Deng, Mengjiao Zhang, Xianchun Chen, Ximing Pu, Xiaoming Liao, Zhongbing Huang, Guangfu Yin

**Affiliations:** * College of Materials Science and Engineering, Sichuan University, Chengdu 610064, PR China

**Keywords:** akermanite, PLGA, composite scaffolds, solvent self-proliferating process

## Abstract

Desirable scaffolds for tissue engineering should be biodegradable carriers to supply suitable microenvironments mimicked the extracellular matrices for desired cellular interactions and to provide supports for the formation of new tissues. In this work, a kind of slightly soluble bioactive ceramic akermanite (AKT) powders were aboratively selected and introduced in the PLGA matrix, a novel l-lactide modified AKT/poly (lactic-*co*-glycolic acid) (m-AKT/PLGA) composite scaffold was fabricated via a solvent casting-particulate leaching method improved by solvent self-proliferating process. The effects of m-AKT contents on properties of composite scaffolds and on MC3T3-E1 cellular behaviors *in vitro* have been primarily investigated. The fabricated scaffolds exhibited three-dimensional porous networks, in which homogenously distributed cavities in size of 300–400 μm were interconnected by some smaller holes in a size of 100–200 μm. Meanwhile, the mechanical structure of scaffolds was reinforced by the introduction of m-AKT. Moreover, alkaline ionic products released by m-AKT could neutralize the acidic degradation products of PLGA, and the apatite-mineralization ability of scaffolds could be largely improved. More valuably, significant promotions on adhesion, proliferation, and differentiation of MC3T3-E1 have been observed, which implied the calcium, magnesium and especially silidous ions released sustainably from composite scaffolds could regulate the behaviors of osteogenesis-related cells.

## Introduction

To overcome the shortages of bone graft transplantation, tissue engineering has been considered as a feasible approach to repair and reconstruct the bone defects caused by wounds or bone diseases [1]. As a momentous component of bone tissue engineering, scaffolds served as the temporary platform which provided a suitable space and microenvironment to allow cell adhesion, migration and proliferation, to deliver the biochemical factors, to enable diffusion of nutrients and metabolites, and to support new tissue formation. Primarily, scaffolds should possess adaptive biomechanical properties and three-dimensional interconnected pore networks. It has been described that pore sizes ranging between 300 and 400 μm were most suitable for the regeneration of bone tissues and high porosities were able to promote cell migration and vascularization of ingrown tissue [2–4]. More importantly, the desirable scaffolds for bone tissue regeneration should be osteoinductive and biodegradable, and the degradation products should have no adverse effects on seeded cells and neighboring tissues [5]. All these properties would be closely related to the processing methods and the matrix materials.

Numerous techniques have been employed to fabricate scaffolds, such as solvent casting-particulate leaching, gas foaming, phase separation and electrostatic spinning [6–9]. Solvent casting-particulate leaching (SC-PL) method was most commonly used owing to simple operation, and the pore size and the porosity of scaffolds could be well controlled by the particle size and the amount of the incorporated salt particles. However, the traditional SC-PL method tended to cause the isolated and heterogeneously distributed pores, resulting from the high viscosity of polymer solution and the poor contact degree of salt particles in polymer solution, as well as the different apparent densities between salt particles and polymer solution [10].

Even much greater concern was the matrix materials for fabrication of scaffolds. Nowadays, a wide variety of composite materials have been being investigated to aspire after the controllable physical and chemical properties as well as to construct a favorable microenvironment *in vivo* for regeneration of bone tissues. PLGA as the copolymers of PGA and PLA has been widely utilized as biodegradable and biocompatible material in many fields, such as drug-delivery, orthopedic fixation devices and construction of tissue engineering scaffolds [11–13]. It could be facilely molded into irregular even more complex shapes to match with defect sites, and its degradation rate could be regulated by adjusting the amount of PLA and PGA units to accommodate to the tissue regeneration rate. However, the poor bioactivity, the acid degradation products and the suboptimal mechanical properties of PLGA scaffolds have limited its extensive application in bone tissue engineering [14].

It has been much prevalent to combine bioactive inorganic fillers with biodegradable polymers to fabricate composite scaffolds. In such scaffolds, the appropriate inorganic fillers could not only reinforce porous structures, but also play a vital role in promoting cell adhesion, proliferation and osteogenic differentiation [15]. Furthermore, bioactive fillers should be capable of neutralizing the acidity of polymer degradation products to avoid aseptic inflammatory response, and facilitating the formation of bone-calcium phosphate interface layer [16]. However, there was a common problem associated with inorganic fillers/polymer composite via directly mixing without else treatment, which the incorporated fillers would not form a close interface with polymer matrix and would not homogeneously dispersed, due to the poor interface compatibility resulting from the high surface energy of fillers and low surface energy of polymer [17].

A number of inorganic materials have been proved to be bioactive, such as hydroxyapatite (HA), calcium phosphate (β-TCP), wollastonite (CS), silicate-based bioglass® and some Ca–Mg–Si-containing bioceramics [18–23]. As a member of Ca–Mg–Si-containing bioceramics, akermanite (AKT) (Ca_2_MgSi_2_O_7_, AKT) has received significant attention owing to its superior apatite-mineralization and osteoinduction ability by the release of soluble ionic products [24, 25]. The slightly soluble AKT could release calcium ions, magnesium ions and silidous ions in aqueous solutions to create an alkalescent microenvironment. And studies *in vivo* have indicated that AKT possessed more stronger abilities of promoting bone regeneration and angiogenesis when compared with traditional β-TCP [26, 27].

In this work, a novel l-lactide modified AKT/poly (lactic-*co*-glycolic acid) (m-AKT/PLGA) composite scaffold was fabricated via a solvent casting-particulate leaching method improved by solvent self-proliferating process, in which the AKT powders were aboratively selected to reinforce the scaffolds, to supply an alkalescent microenvironment, and especially to promote the osteogenesis instead of the growth factors. Firstly, AKT powders were modified with the l-lactide to improve the interface compatibility with PLGA, and then introduced in the PLGA matrix. Subsequently, the solvent casting-particulate leaching method was technically improved by the solvent self-proliferating process and then applied to fabricate the m-AKT/PLGA composite scaffolds with interconnected and homogenously distributed pores. Furthermore, the influences of m-AKT powders on the scaffold performances and the cellular behaviors of MC3T3-E1 cells were systemically investigated.

## Materials and methods

### Preparation and surface modification of AKT powders

AKT powders were synthesized using calcium nitrate tetrahydrate (Ca(NO_3_)_2_·4H_2_O), magnesium nitrate hexahydrate (Mg(NO_3_)_2_·6H_2_O) and tetraethyl orthosilicate ((C_2_H_5_O)_4_Si, TEOS) as raw materials by sol–gel method [24]. Powders were wet ground by a planetary ball mill using ethanol as liquid medium. After that, ethanol was replaced by deionized water. AKT powders were received after freeze-drying. Powders were modified with l-lactide to improve the compatibility with PLGA. 3 g l-lactide (LLA) was dissolved in 20 ml dimethylbenzene, and the homogeneous LLA solution was drop slowly into AKT dimethylbenzene suspension (3 g AKT powders with 10 ml dimethylbenzene) at 90°C. The grafting polymerization of LLA on AKT surfaces was carried out with 0.1 wt% Sn(Oct)_2_ as a catalyst at 120°C for 48 h under gentle stirring and the protection of nitrogen. Finally, the modified AKT (m-AKT) powders were collected by filtration, and then, rinsed with chloroform and distilled water three times, and vacuum-dried at 60°C. The m-AKT powders were characterized by Laser Particle Size Analyzer (LPSA; Mastersizer 3000E, Malvern, UK), X-ray diffraction (XRD; X’Pert MPD 3 kW, Philips, The Netherlands), and Fourier transform infrared spectrometer (FTIR; Nicolet 6700, Thermo Scientific, Germany).

### Fabrication of porous scaffolds

Scaffolds were fabricated by the combination of solvent casting, solvent self-proliferating and salt particle leaching method. First, a certain amount of PLGA powders (75/25) (*M*_w_ = 132 kDa, *M*_n_ = 84 kDa, purchased from Institute of Medical Instrument Tsinan, Shandong Province, China) were dissolved in chloroform to form homogeneous solution (0.2 g/ml), and the solution was poured into m-AKT chloroform suspension (0.4 g/ml) under continuous stirring for 2 h. Then sodium chloride (NaCl) particles (particle size ranging from 300 to 400 μm) were added into the suspension and stirring until the mixture became viscous slurry. Subsequently, the slurry was slowly dropped into a porous cylindrical mesh (Φ 12 mm) which was immersed in anhydrous ethanol under moderate agitation. Uncompacted composite formed after 1 h, then loaded into a mold (inner diameter Φ12 mm) and compressed at 10 MPa for 5 min. Finally, the compressed composite was soaked in fast-agitated distilled water containing 20% (v/v) ethanol for 24 h to leach out the NaCl particles, and the m-AKT/PLGA porous scaffold was obtained after air-dried.

To confirm the suitable NaCl particles dosage in which the scaffolds exhibited desired porosity and mechanical strength for bone tissue engineering, different amounts of NaCl particles were added to fabricate the pure PLGA scaffolds (mass ratio of NaCl to PLGA, 8:1, 7:1, 6:1, 5:1 and 4:1). Based on the results, different composite scaffolds (mass ratio of m-AKT to PLGA, 1:9, 1:4 and 2:3, marked as C10, C20 and C40, respectively) were prepared to investigate the effects of m-AKT content on the properties of scaffolds (optimal mass ratio of NaCl to PLGA and m-AKT). All the scaffolds were fabricated with the same processes. The only difference was the mass ratio of NaCl to PLGA for pure PLGA scaffolds, and the mass ratio of m-AKT to PLGA for composite scaffolds.

### Characterization of scaffolds

The section morphology of scaffolds was observed by scanning electron microscope (SEM; JSM-5900LV, Hitachi, Japan) coupled with an energy-dispersive spectrometer (EDS). The porosity of scaffolds was measured by Archimedes immersion technique as our previous research described [28]. In brief, cylindrical scaffold (*Ф* 12 × 3 mm^2^) with dried mass $G_{1}$was immerged into a porosity analyzer (DXR, Xiangtan Apparatus Plant, China). Ethanol was used as the liquid medium, and penetrated into the pores of the whole scaffold under vacuum. Next, scaffold was removed from the ethanol. Wiped away the ethanol on the surface of sample, and the sample was weighed as${\;G}_{2}$. The porosity was calculated by the following equation: Porosity$(\%)=\left(G_{2}-G_{1}\right)/\rho \pi R^{2}h\times 100\%$, where ρ  is the density of ethanol, *R* is the radius of scaffold and h is the height of scaffold. The compressive strength of cylindrical scaffolds (*Ф* 12 × 3 mm^2^) was determined by a mechanical testing apparatus (AG-IC 50KN, Shimadzu, Japan). Briefly, scaffold was placed between two parallel plates and compressed with a constant deformation rate of 1 mm/min. The compressive strength of scaffold was defined when the specimen was compressed to 30% of its original thickness.

### *In vitro* degradation of scaffolds

Pure PLGA scaffolds (C0), composite scaffolds with m-AKT content of 20 wt% (C20) and 40 wt% (C40) were selected to carry out the degradation experiments by incubating the samples (*Ф* 12 × 3 mm^2^) in phosphate-buffered saline (PBS, pH = 7.4) at 37°C. Each scaffold was weighed as $W_{d}$ (dried mass) and placed in a polyethylene tube. The amount of PBS using as a degradation medium was 50 times${\;W}_{d}$. All tubes were evacuated for 10 min to ensure the scaffolds were immersed in PBS. Then, the tubes were sealed and placed in a shaking water bath with 120 rpm at 37°C up to 12 weeks. Three tubes of each kind of scaffolds were selected out for characterization every week. The degradation medium was collected to measure the pH value variation with a pH meter (PHS-3 C, Rex, China). The scaffold taken out from the tube was washed with deionized water and vacuum-dried for 24 h then weighed as${\;W}_{t}$. The weight loss of the scaffold was calculated as following expression: Weight loss$(\%)=\left(W_{d}-W_{t}\right)/W_{d}\times 100\%$. And the compressive strength of scaffolds was also measured.

### *In vitro* bioactivity of scaffolds

*In vitro* bioactivity of the cylindrical scaffolds (*Ф* 12 × 2 mm^2^) was assessed with the mineralization of hydroxyapatite by immersing the samples in simulated body fluid (SBF, pH = 7.4) at 37°C. The preparation of SBF was according to our previous research [28]. Scaffolds C0, C20 and C40 were soaked in SBF solution in a polyethylene container at 37°C (the ratio of SBF volume to scaffolds mass was 200 ml/g). After immersion for 1, 3, 5, 7 and 14 days, the Ca, P, Si and Mg ionic concentration, respectively, of SBF in containers were analyzed by inductively coupled plasma optical emission spectroscopy (ICP-OES; Vista-MPX, Varian, USA). After soaked for 2 weeks, the scaffolds were removed from SBF and rinsed with distilled water, then vacuum-dried. The surface morphology of the scaffolds after immersion, as well as the chemical composition at some specific sites, was determined by SEM, EDS and XRD.

### Cell culture

MC3T3-E1 cells (purchased from the Shanghai Institute of Biochemical and Cell Biology) were cultured in a flask with Dulbecco's modified Eagle's medium (DMEM) containing 10% (v/v) fetal calf serum at 37°C in a humidified atmosphere with 5% CO_2_. The culture medium was replaced every two other days. When reaching 70–80% confluence, the cells were harvested from the bottom of culture flask with 0.25% Trypsin-EDTA and used in the subsequent study.

### Cytoskeletal and morphology of MC3T3-E1 cells on scaffolds

The scaffolds (three-dimensional size 8 × 8 × 2 mm^3^) were sterilized in 75% ethanol for 2 h and then were air-dried in a UV sterilized super clean bench for a whole day. Before cell seeding, the scaffolds were washed thrice with sterile PBS and then placed into 24-well plates. MC3T3-E1 cells were seeded onto the scaffolds at an initial density of 1 × 10^4^ cells per scaffold. The culture medium was removed and the scaffolds were rinsed twice with PBS after cultured for 3 days. For confocal microscopy imaging, the cells on scaffolds were fixed with 4% paraformaldehyde for 30 min. The F-actin and nuclei of cells were, respectively, stained with rhodamine phalloidin for 20 min and 4′,6-diamidino-2-phenylindole for 10 min. Finally, cells were rinsed with PBS two times. The confocal images were taken using a fluorescence microscope (Leica TCS SP5, Germany). For morphology observation, cells were fixed 2 h with 2.5% glutaraldehyde. Next, the fixed cells were dehydrated by a graded series of ethanol (30%, 50%, 70%, 80%, 90%, 95% and 100%) for 15 min. The cell morphology was observed by SEM.

### Cell proliferation of MC3T3-E1 cells on scaffolds

The proliferation of MC3T3-E1 cells cultured on scaffolds (8 × 8 × 2 mm^3^) was determined by the quantitative MTT (3-(4,5-dimethylthiazol-2-yl)-2,5-diphenyl tetrazolium bromide) assay. In brief, sterilized scaffolds were placed in 24-well plates, and cells were seed onto scaffolds (1 × 10^4^ cells per scaffold) then cultured. At the set time points, the Ca, Mg and Si ionic concentrations of the cell culture medium were analyzed by ICP-OES, and the scaffolds were irrigated with PBS two times to remove the residual culture medium. Five hundred microlitre of MTT solution (0.5 mg/ml) was added to each well to immerse the scaffold and then incubated for 4 h. Subsequently, the MTT solution was replaced by 500 μL dimethyl sulfoxide (DMSO) to dissolve the formazans crystals completely for 15 min. The formazan solution of each sample was transferred to the wells of a 96-well plate (100 μL per well) and the absorbance at 490 nm was measured using a microplate reader (EL × 800, BIO-TEK, Atlanta, GA).

### Evaluation of osteogenic differentiation of MC3T3-E1 cells on scaffolds

MC3T3-E1 cells were cultured on the scaffolds C0, C20 and C40 at the same conditions described above. After incubation for different time, the culture medium was removed and the cells on scaffolds were rinsed two times with PBS followed by trypsinization. The cell lysate products were obtained by three cycles of freezing and thawing [29]. ALP activity was assayed by commercially available test kits as described by the manufacture. To assess Col I and OCN expression, Col I and OCN contents in cell lysates were measured using ELISA assay kits according to the instructions from the manufacturer, respectively. All test kits were purchased from Jiancheng Institute of Biotechnology (Nanjing, China).

### Statistical analysis

All the data were presented as mean ± standard deviation (SD) from at least three individuals. Analysis of the results was performed by one-way ANOVA using SPSS18.0 software. A *P* values <0.05 was considered statistically significant.

## Results and discussion

### Characterization of AKT powders

**Figure 1.**
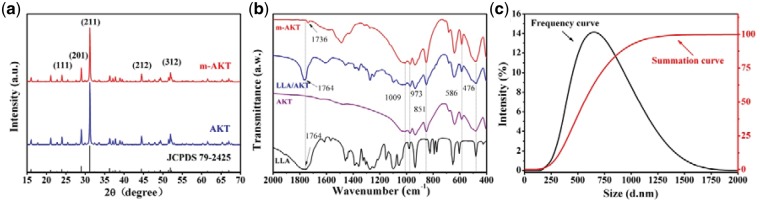
Characterizations of powders. (a) XRD spectra. (b) FTIR pattern. (c) Particle size distribution

The AKT powders were synthesized and modified by LLA. The XRD spectra of AKT and modified AKT powders (m-AKT) are shown in Fig. 1a. It was obvious that AKT and m-AKT diffraction peaks existed at 2*θ* = 23.93°, 28.90°, 31.12°, 44.43° and 51.88° corresponded to the (1 1 1), (2 0 1), (2 1 1), (2 1 2) and (3 1 2) crystal planes of AKT (Ca_2_MgSi_2_O_7_) registration in the JCPDS database (Standard card no. JCPDS 79-2425), respectively. These peaks were well complied with the AKT. The FTIR pattern of AKT powders modified before and after is shown in Fig. 1b. Peaks at 1009 cm^−1^, 973 cm^−1^, 851 cm^−1^, 586 cm^−1^ and 476 cm^−1^ showed in AKT, LLA/AKT (physical mixture of LLA and AKT) and m-AKT spectra belong to the characteristic absorption peaks of AKT [30, 31]. It could be observed that spectra of LLA and LLA/AKT displayed a characteristic carbonyl peak at 1764 cm^−1^, and m-AKT spectra showed an absorption peak at 1736 cm^−1^ which might be attributable to the carbonyl group of the polyester originated from the polymerization of LLA on the surface of AKT powders [17]. These results indicated that m-AKT powders were successfully prepared without obvious structural diversification before and after modification. The particle size of m-AKT powders were estimated to be 681.4 ± 247.8 nm and presented normal distribution according to Fig. 1c.

### Fabrication and characterization of scaffolds

**Figure 2.**
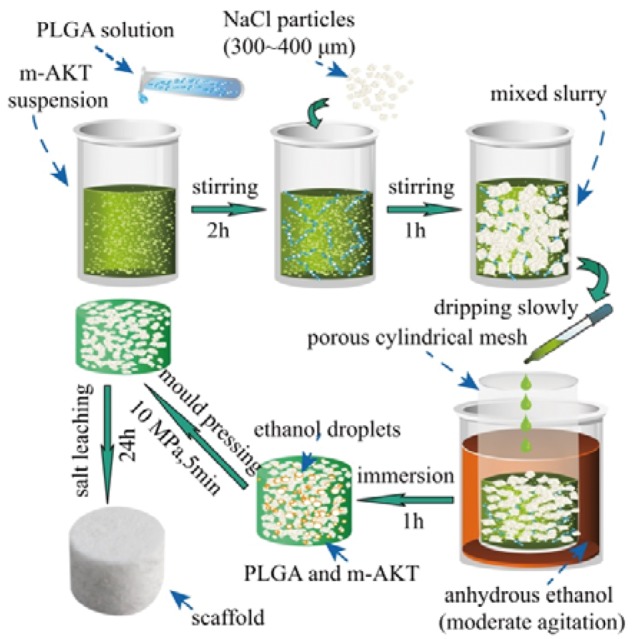
Steps of the improved solvent casting-salt particle leaching method

All the scaffolds were fabricated by the combination of solvent casting, solvent self-proliferating and salt particle leaching method. The production process flow is shown in Fig. 2. Solvent casting-salt particle leaching (SC-PL) was one of the most popular techniques to fabricate porous scaffolds. However, the traditional SC-PL method tended to cause isolated pores resulting from the high viscosity of polymer solution as well as the poor contact degree of salt particles in polymer solution. In most cases, the salt particles were heterogeneously distributed in the polymer due to the different apparent densities between salt particles and polymer solution [10]. In this work, the fabrication method was improved by the solvent self-proliferating process based on the traditional SC-PL method. The mixed slurry (using chloroform as solvent) of PLGA, m-AKT and NaCl particles was slowly dropped to a porous cylindrical mesh immersed in slow stirring anhydrous ethanol. Because the chloroform in slurry might easily diffuse into ethanol, the PLGA in dripped slurry would be quickly supersaturated, resulted in the fast nucleation and semi-solidification of PLGA on the surface of m-AKT particles and NaCl particles, and the semi-solidified PLGA could prevent NaCl particles from subsiding. As the results, NaCl particles would be homogenously distributed in the PLGA matrix, and an uncompacted composite formed after 1 h immersion. After that, the composite was compressed under 10 MPa for 5 min to increase the direct contact between NaCl particles and to impel the semi-solidified PLGA matrix to fill the gaps or space which was occupied by ethanol droplets. Finally, the composite was soaked in fast-agitated distilled water containing 20% (v/v) ethanol to dissolve NaCl particles. As we all known, ethanol was easily to penetrate into PLGA matrix, which have a softening effect on the polymer, resulting in the chain mobility increase. This would facilitate the water molecules to leach out NaCl particles.

**Figure 3.**
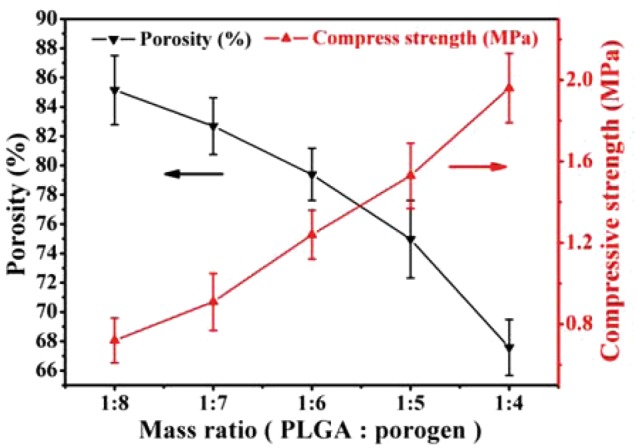
Effect of porogen dosage on the porosity and compressive strength of pure scaffolds

On the other hand, the porosity, pore interconnectivity and mechanical strength of the scaffolds were closely related to the salt particles dosage. Therefore, the effects of the salt particles dosage on the porosity and mechanical strength of pure PLGA scaffolds were investigated to determine the appropriate salt particles dosage in composite scaffolds used for bone tissue engineering. The porosity of the pure PLGA scaffolds was ranging from 67.6 ± 1.9–85.2 ± 2.3%, which increased with the increase of the dosage of porogens. On the contrary, the compressive strength of the pure scaffolds decreased from 1.96 ± 0.17–0.72 ± 0.11 MPa (shown in Fig. 3). Researches have shown that porosity approximate or larger than 80% would be propitious to promote cell migration and vascularization of ingrown tissue [3, 4]. Our results showed that the pure PLGA scaffolds exhibited with the compressive strength of 1.24 ± 0.12 MPa and the porosity of 79.4 ± 1.8% when the mass ratio of porogens to PLGA was 6:1, and this ratio was selected and fixed in the fabrication of composite scaffolds. The scaffolds not only have high porosity but also possess moderate compressive strength. However, the compressive strength of scaffolds needed to be enhanced for bone tissue engineering applications. In order to reinforce the scaffold matrix and to endow the scaffold with osteoinductivity, m-AKT powders were introduced to fabricate composite scaffolds (a mass ratio of porogen to PLGA and AKT powders was fixed as 6:1).

**Figure 4.**
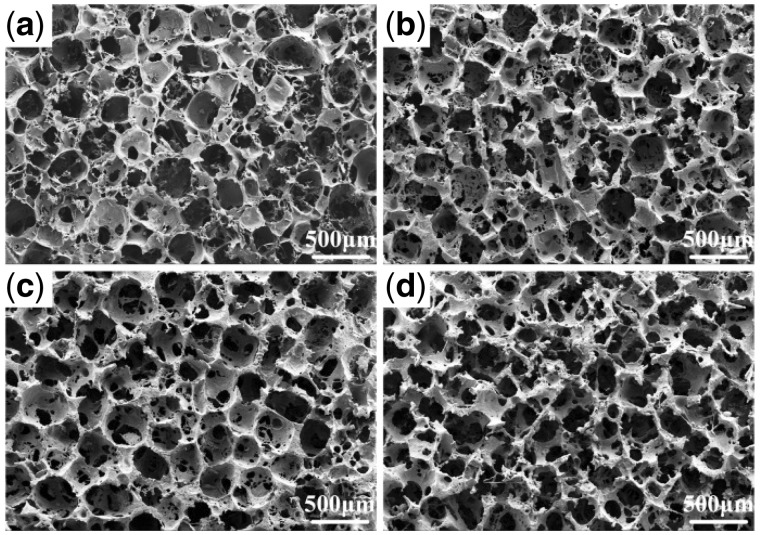
SEM images of the cross section of scaffolds. (a) C0. (b) C10. (c) C20. (d) C40

The cross section morphology of scaffolds was observed by SEM and is shown in Fig. 4. Pores formed after the salt particles were leached out. The vast majority of cavities were irregular polygonal in shape with the size of 300–400 μm, and were distributed homogenously in scaffolds and interconnected by some smaller holes (sized 100–200 μm) in the walls. This desired pore structure could indirectly verify the improvement of fabrication method by the solvent self-proliferating process. It could be also found that the walls of pure PLGA scaffolds (C0) were relatively intact comparing with that of the composite scaffolds, and the number of small holes on the wall of composite scaffolds increased and the size trended to bigger with the increase of m-AKT contents, especially, the walls in the scaffolds C40 were almost damaged. It might be because the apparent density of m-AKT powder was larger than PLGA, and the same weight of composite (m-AKT powders and PLGA) occupied smaller volume than PLGA, resulting in too large volume ratio of the porogens.

**Figure 5.**
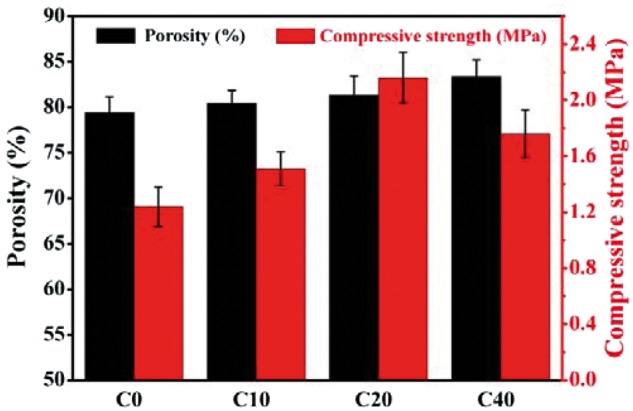
Effect of m-AKT content on the porosity and compressive strength of scaffolds

Porosity and compressive strength of the scaffolds are shown in Fig. 5. The porosity of composite scaffolds was a little higher than pure PLGA scaffolds owing to the larger volume ratio of porogens. The compressive strength of composite scaffolds gradually became higher than that of pure PLGA scaffolds with the increase of m-AKT contents via the reinforcement of m-AKT powders on the PLGA matrix. When the content of m-AKT was 20%, the compressive strength of scaffolds reached to 2.16 ± 0.18 MPa, and the porosity was 81.3 ± 2.1%. However, while the amount of m-AKT increased to 40%, the compressive strength of scaffolds C40 was rapidly dropped down to 1.76 ± 0.17 MPa, only a little higher than scaffolds C10 (1.51 ± 0.12 MPa) and much lower than scaffolds C20. The results indicated that the reinforcement effect of m-AKT powders on scaffolds would be seriously decreased due to the damage of wall when the addition of m-AKT powders exceeded a certain limit.

**Figure 6.**
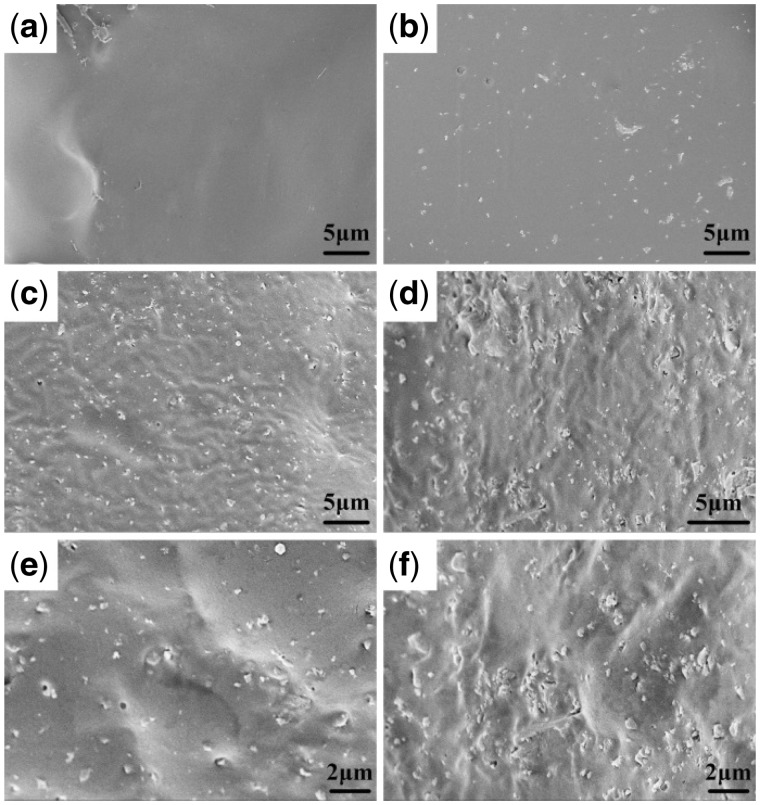
SEM images of the ruptured section of scaffolds. (a) C0. (b) C10. (c), (e) C20. (d), (f) C40

It has been found that the volume fraction and the distribution of the incorporated inorganic particles would be the main factors determining the level of reinforcement [32]. In order to further understand the variation rules of strength with the addition of m-AKT powders, the textures, configurations, as well as the m-AKT distributions in PLGA matrix were observed by SEM imaging for the ruptured section of scaffolds (shown in Fig. 6). The m-AKT powders in scaffolds C10 and C20 were uniformly dispersed in PLGA matrix, and the agglomeration of m-AKT particles was negligible. In particular, the incorporated inorganic particles contacted closely with polymer matrix without notable interfaces due to the strong linkages between the PLGA matrix and the LLA grafted on the surface of m-AKT powders. However, obvious agglomerations of m-AKT particles were observed and the particles were unevenly distributed in the matrix resulting from the overdosage of m-AKT powders for the scaffolds C40. Both the minor linkage between matrix and surfaces of aggregates and the slack interfacial contact between m-AKT powders inside aggregates made the m-AKT particles to be relatively slid, even led to the destruction of scaffold integrity under the external force. As a result, the compressive strength of scaffolds reduced pronouncedly.

### *In vitro* degradation of scaffolds

**Figure 7.**
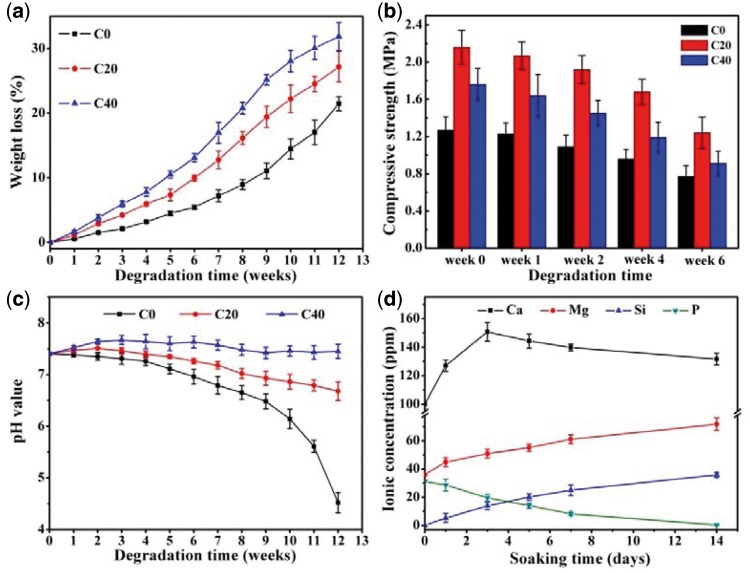
(a) Weight loss of scaffolds. (b) Compressive strength of scaffolds. (c) pH value variation of the degradation medium. (d) Ca, Mg, Si and P ionic concentrations in SBF after scaffolds C40 were immersed for different periods

The degradation of m-AKT/PLGA composite scaffolds in PBS primarily involved the fracture of PLGA molecular chain and the dissolution of AKT. Macroscopically, the weight loss, the pore enlargement, the strength reduction and other phenomena would be emerged in the degradation process. The weight loss ratio of scaffolds is exhibited as in Fig. 7a. The weight loss of all groups increased progressively during the whole degradation period. At the end of 12th weeks, the weight loss of group C0 (WL = 21.5%) was lower than that of group C20 (WL = 27.2%) and group C40 (WL = 31.8%). For group C0, the rate of weight loss was slow at the beginning, and then sharply increased after eighth weeks. During the whole degradation period, the weight loss of group C20 and C40 was higher than that of group C0, and the weight loss speed of group C20 and C40 gradually tended to slow, especially for group C40. Fig. 7b shows that the compressive strength of all scaffolds decreased with the immersion time increasing, and the compressive strength of composite scaffolds was higher than that of pure scaffolds all the time owing to the enhancement effect of m-AKT phase. It could be seen from Fig. 7c that the pH of group C0 presented a slight decrease during the first 9 weeks, and then decreased dramatically until the experiment ended at 12th weeks (pH = 4.52). However, the pH of groups C20 and C40 appeared mild increase in the first few weeks followed by a light decrease. At the end of several weeks, the pH value of group C40 and C20 gradually tended to stabilize at 7.45 and 6.68, respectively. Literatures had suggested that the hydrolysis of the PLGA ester group mainly happened on the surface of PLGA scaffolds since the poor hydrophilic at the beginning. For composite scaffolds, the hydrolysis could occur simultaneously on surface as well as in interior for the hydrophilic fillers promoting hydrone to permeate through PLGA matrix [33]. With degradation continue, the previous acid degradation products residual inside the scaffolds accelerated the degradation of scaffolds C0 duo to the autocatalytic effect, and the degradation products gradually diffused into surrounding media result in the weight loss increase and pH decrease sharply. The researches of H. Li et al have indicated that the incorporation of some types of alkalinity inorganic fillers (such as BG and CS) in polymer scaffolds could weaken the autocatalytic effect and compensate the pH decrease on account of the release of Ca and Si ionic products.[20, 34]. Previous studies have also indicated that alkalinity AKT powders possess slight dissolving capacity, and could release Ca, Mg and Si ionic products resulting in the pH increase of degradation medium [25, 27]. Fig. 7d reveals that the Ca, Mg and Si ionic concentration increased in SBF after scaffolds C40 immersed for different periods. These results above indicated that the dissolvable ionic products of m-AKT powders in composite scaffolds could neutralize the acidic degradation products released by PLGA to improve the acidic degradation environment.

### *In vitro* bioactivity of scaffolds

**Figure 8.**
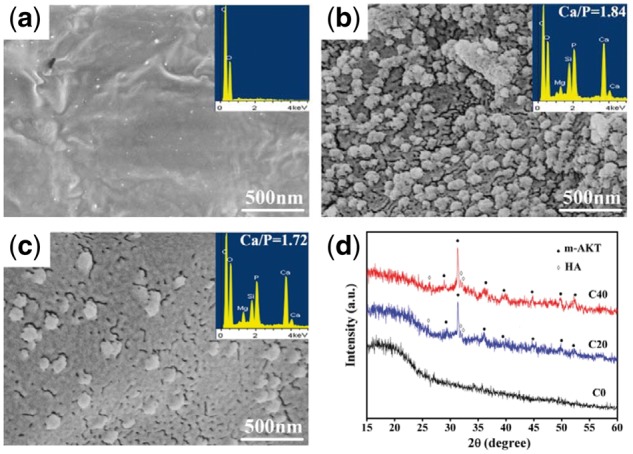
SEM images of surface morphology and EDS of scaffolds C0 (a), C20 (b), and C40 (c). (d). XRD pattern of scaffolds after soaked in SBF for 2 weeks

The mineralization of hydroxyapatite on the material surface in simulated physiological environments has been regarded as the important indicator of the bioactivity of bone repair materials. The apatite-mineralization ability on m-AKT/PLGA composite scaffold has been investigated via the surface morphology observation and chemical composition analysis on the scaffolds soaked in SBF for 2 weeks as well as the ionic concentration changes in SBF after scaffolds C40 immersed for different periods (shown in Figs. 8 and 7d, respectively). The surface morphology of scaffolds C0, C20 and C40 after soaked in SBF for 2 weeks is shown in Fig. 8a–c. The pure PLGA scaffold C0 exhibited a smooth surface without any observable mineralized product. However, large amounts of spherical mineralized nanoparticles were formed and distributed uniformly on the surface of scaffold C20. As for the scaffold C40, the surface was fully covered by a thin film of mineralized nanoparticles, and a small number of large particles were observed. The surficial chemical composition analysis (EDS) clearly showed that Ca and P peaks were detected on the surface of scaffolds C20 and C40 in comparison with scaffolds C0, and the Ca/P molar ratio were, respectively, 1.84 and 1.72, in close proximity to the stoichiometric of perfect hydroxyapatite 1.67. Fig. 8d shows the chemical components of scaffolds after scaffolds immersed for 2 weeks, and the characteristic peaks of hydroxyapatite were observed on scaffolds C20 and C40, but not found on scaffolds C0. The results of SEM observation, EDS analysis and XRD pattern demonstrated the apatite-mineralization ability of m-AKT/PLGA composite scaffold, and the decreases of P and Ca ionic concentrations in SBF could also indicate the formation of calcium phosphate precipitates.

Filipowska *et al*. [35] found that the surfaces of sol-gel bioactive glass/PLGA composite scaffolds were completely covered by spherical calcium phosphate deposits after the scaffolds soaked in SBF for 2 weeks. Previous studies have also indicated that AKT possess superior apatite-mineralization ability in SBF and could induce the formation of round or lath-like apatite crystallites [24, 25]. In this study, the nano-apatite particles were found both on the surfaces of scaffolds C20 and C40 after soaking 2 weeks. To some extent, the mechanism of apatite formation on m-AKT/PLGA composite scaffolds might be similar with bioactive glass [36]. When m-AKT/PLGA scaffolds were soaked in SBF, the H^+^ and H_3_O^+^ attacked Ca–O, Mg–O ionic bonds and Si–O covalent bonds. As the bond energy of Ca–O bond was much lower than Mg–O and Si–O bond, Ca–O bonds were easy to break under the hydrolysis. Lots of H^+^ took the places of Ca^2+^ and then the Ca^2+^ diffused into SBF resulting in the formation of silanol groups and negative charge layer on the surfaces of scaffolds. Under the action of electrostatic field force, the Ca^2+^ concentration around composite scaffolds surface gradually became higher than that in SBF. When the combination rate of Ca^2+^ and phosphate groups (PO3- 4) became faster than their dissolution velocity, the Ca^2+^ would unite with PO3− 4 to form nucleation of nano-apatite particles and grew up. It was obvious that the thin film consisted of numerous spherical nano-apatite particles on the scaffolds C40, and many isolated spherical nano-apatite particles were distributed on scaffolds C20. It might be attributed to that more silanol groups (nucleation sites) exposed on the surfaces of scaffolds C40 resulted in larger nucleation density with the increase of content of m-AKT, and then large numbers of crystal nucleus grew up and finally formed the overlying strata of apatite. It had been reported that apatite layer similar to the inorganic mineral phase of bone on the surface of the material provided an ideal environment for six cellular reaction steps that included colonization by osteoblasts, followed by proliferation and differentiation of the cells, and the formation of apatite could combine with some extracellular matrix ingredient secreted by osteoblasts to form new bone that had a mechanically strong bond to the implant surface [37]. The presence of apatite particles on composite scaffolds indirectly indicated that the incorporation of m-AKT powders could improve the bioactivity of composite scaffolds.

### MC3T3-E1 cellular behaviors on scaffolds *in vitro*

#### Cell attachment and cell morphology on scaffolds

**Figure 9.**
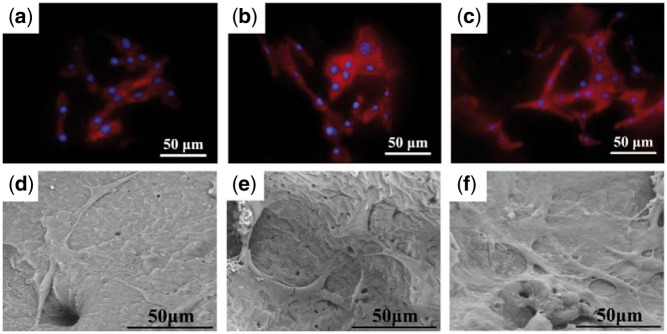
Confocal images and SEM images of MC3T3-E1 cells after three days culture on scaffolds: (a), (d) C0. (b), (e) C20. (c), (f) C40

MC3T3-E1 cells were cultured on scaffolds C0, C20 and C40 for 3 days, and the cytoskeletal organization and the morphology of cells are shown in Fig. 9. It can be seen that cells adhered to the surface of all scaffolds. A large proportion of cells on scaffold C0 presented the elongated morphology, but the cells on scaffolds C20 and C40 mainly exhibited the polygonal shape and the larger volume. This indicated that cells spread and grew better on the composite scaffolds. The surface characteristics of scaffolds have been known to significantly affect cell adhesion and spreading. Previous literatures have shown that the rough and hydrophilic surface of biomaterials were favorable to the interactions between the materials and cells [38]. The surfaces of prepared composite scaffolds (shown in Fig. 6) appeared with sags and crests due to the impaction of m-AKT powders, and the existence of m-AKT powders in scaffolds could improve the hydrophilic of scaffolds.

### Proliferation of MC3T3-E1 cells on the scaffolds

**Figure 10.**
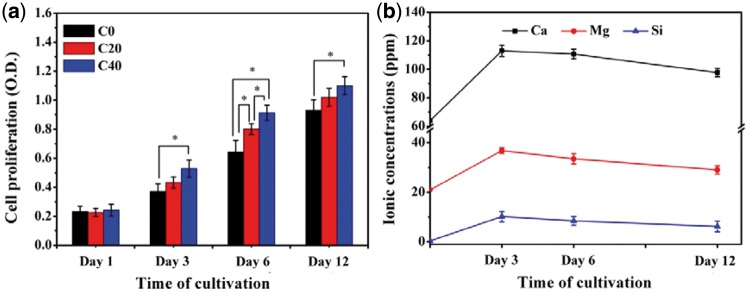
(a) Proliferation of MC3T3-E1 cells on caffolds. (b) Ionic concentrations of cell culture medium of C40 group

Osteoblast proliferation played an important role in the process of bone formation. The proliferation of MC3T3-E1 cells on scaffolds C0, C20 and C40 was quantitatively evaluated using MTT assay as an indicator of cells number after the cells were cultured for different time. The optical density (OD) absorbance values for different groups are shown in Fig. 10a. It was quite clear that the OD value of all groups increased with culture time, and this indicated that all the scaffolds possessed good cytocompatibility. Fig. 10a also illustrates that there was no significant differences of OD value after 1 day culture, while group C40 and group C20 presented obviously higher OD value than that of group C0 after 3 days culture. Fig. 10b shows the Ca, Mg and Si ionic concentrations of cell culture medium of group C40, and the ionic concentrations of cell culture medium were much higher than that of the initial culture medium on account of the dissolve of m-AKT powders in composite scaffolds during the whole cultivation process. The results proved that the introduction of m-AKT could promote the proliferation of MC3T3-E1 cells on the composite scaffolds, and the promotion effects would gradually revealed with the release of involved ions. Previous studies have suggested that Si was an indispensable element for the reconstruction and calcification of bone tissue, and the existence of silidous ions released from bioactive glass were in favor of the proliferation of cells [26,39,40]. In this study, the dissolution of m-AKT in composite scaffolds not only increased the ionic concentration of Ca^2+^ and Mg^2+^ but also supplied the silidous ions which were originally barely existed in the culture media. In addition, the releasing of Ca and Mg ions made the culture medium slightly alkaline which were propitious to the growth of the basophile MC3T3-E1 cells. Therefore, it might be considered that Ca, Mg and Si-rich environment was mainly responsible for the high proliferating rate of composite scaffolds.

### Osteogenic differentiation of MC3T3-E1 cells on the scaffolds

**Figure 11.**
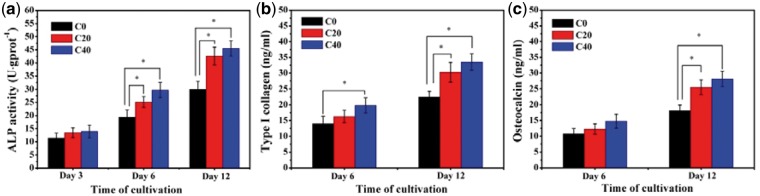
Osteogenic differentiation of MC3T3-E1 cells on scaffolds: (a) ALP activity, (b) type I collagen, and (c) osteocalcin

Osteogenic differentiation was known as the critical process closely related to the mineralization of extracellular matrix for bone regeneration. Alkaline phosphatase activity was widely recognized as the determinate marker of early differentiation of osteoblasts because the main physiological functions of alkaline phosphatase (ALP, a glycoprotein external to the cell membrane) were involved in the hydrolyses of the organic alkaline and the inorganic pyrophosphate to increase local concentration of orthophosphate and relieve the inhibition to bone salt formation for bone mineralization [41]. Type I collagen (Col I) was the major extracellular matrix protein, accounting for approximately 85% of the protein in bone synthesis. Type I collagen synthesis promoted osteoblastic differentiation and mineralization, and increased as the osteoblastic differentiation proceeded [42]. Osteocalcin (OCN) was associated with bone mineralization owing to its ability to bind calcium and hydroxyapatite, and it was also one of the important indicators of the osteogenic differentiation, which was usually synthesized only by mature osteoblast-like cells in the extracellular matrix of bone tissue [43]. In this research, the ALP activity and the expression of osteogenic-related proteins (Col I and OCN) were detected to investigate the effects of m-AKT component in scaffolds on cell differentiation of MC3T3-E1 (shown in Fig. 11). On the whole, the ALP activity (Fig. 11a) for all three groups obviously increased with the culture time. Among them, all groups maintained at a comparatively low level without significant difference for 3 days culture, and group C40 showed a little higher activity than group C20 and significantly higher than group C0 for 3-day culture, but the ALP activity for both group C20 and group C40 were remarkably higher than group C0 with significant differences for 6 and 12 days culture. The expression of Col I (Fig. 11b) and OCN (Fig. 11c) for three groups increased with the time of cultivation, and the protein expression level of group C40 and C20 was much higher than that of group C0 with significant differences after incubation for 12 days. These indicated that m-AKT incorporated in composite scaffolds could promote the differentiation of MC3T3-E1 cells due to the regulative and stimulative effects of the released ionic products. These results were consistent with other previous studies which have shown that the Ca and silidous ions released from calcium silicate-based bioglass and bioceramics could promote osteoblasts proliferation and differentiation [44, 45].

## Conclusion

It has been a consensus that the main functions of scaffold for tissue engineering should be to supply a suitable microenvironment mimicked the extracellular matrix for the desired cellular interactions, and to provide a supporter for the formation of new tissue. The desirable scaffold should be a biodegradable carrier with three-dimensional interconnected porous network and with sufficient mechanical strength. In this work, the fabrication method of scaffolds was improved by the solvent self-proliferating process based on the traditional SC-PL method, and a kind of slightly soluble CaO–MgO–SiO_2_-based bioceramics AKT was introduced into the scaffolds instead of the usually used growth factors. The fabricated scaffolds exhibited a large number of irregular polygonal cavities (300-400 μm in size) which were homogenously distributed in scaffolds and were interconnected by some smaller holes (sized 100–200 μm) in the walls. This three-dimensional interconnected porous network was capable of transporting nutrients and metabolites, and the surfaces with sags and crests due to the impaction of m-AKT powders was conducive to the cell adhesion and spreading. Moreover, the mechanical structure of the porous scaffold was obviously reinforced by the introduction of m-AKT powders. Compared with pure PLGA scaffolds, the compressive strength of m-AKT/PLGA composite scaffolds could be elevated to 2.16 MPa, while the porosity was 81% when the m-AKT content was 20%. Furthermore, the alkaline ionic products released from m-AKT powders could neutralize the acidic degradation products of PLGA so that the degradation rate of scaffolds would be altered via weakening the autocatalytic effect, and the apatite-mineralization ability of scaffolds could be improved. Meanwhile, the slightly alkaline microenvironment resulted by the release of alkaline ionic products might be in favor of the growth of the basophile MC3T3-E1 cells. More importantly, the sustainably released calcium ion, magnesium ion, and especially silidous ions could synergistically regulate behaviors of osteogenesis-related cells, and exhibit significant promotive effects on adhesion, proliferation and differentiation of MC3T3-E1 cells. These results suggested that the m-AKT/PLGA composite scaffold fabricated by the improved solvent casting-particulate leaching method would be a potential candidate for bone tissue engineering.

## References

[1] Langer R. Tissue engineering. Science 1993;260:920–6. 8493529 10.1126/science.8493529

[2] Tsuruga E, Takita H, Itoh H Pore size of porous hydroxyapatite as the cell-substratum controls BMP-induced osteogenesis. J Biochem 1997;121:317–24. 9089406 10.1093/oxfordjournals.jbchem.a021589

[3] Karageorgiou V, Kaplan D. Porosity of 3D biomaterial scaffolds and osteogenesis. Biomaterials 2005;26:5474–91. 15860204 10.1016/j.biomaterials.2005.02.002

[4] Roy TD, Simon JL, Ricci JL Performance of degradable composite bone repair products made via three-dimensional fabrication techniques. J Biomed Mater Res: Part A 2003;66:283–91. 10.1002/jbm.a.10582 12888998

[5] Miron RJ, Zhang YF. Osteoinduction: a review of old concepts with new standards. J Dent Res 2012;91:736–44. 22318372 10.1177/0022034511435260

[6] Mikos AG, Thorsen AJ, Czerwonka LA Preparation and characterization of poly (l-lactic acid) foams. Polymer 1994;35:1068–77.

[7] Mooney DJ, Baldwin DF, Suh NP Novel approach to fabricate porous sponges of poly(D,L-lactic-co-glycolic acid) without the use of organic solvents. Biomaterials 1996;17:1417–22. 8830969 10.1016/0142-9612(96)87284-x

[8] Lo H, Ponticiello MS, Leong KW. Fabrication of controlled release biodegradable foams by phase separation. Tissue Eng 1995;1:15–28. 19877912 10.1089/ten.1995.1.15

[9] Yoshimoto H, Shin YM, Terai H A biodegradable nanofiber scaffold by electrospinning and its potential for bone tissue engineering. Biomaterials 2003;24:2077–82. 12628828 10.1016/s0142-9612(02)00635-x

[10] Sin DC, Miao X, Liu G Polyurethane (PU) scaffolds prepared by solvent casting/particulate leaching (SCPL) combined with centrifugation. Mater Sci Eng C 2010;30:78–85.

[11] Zhou H, Lawrence JG, Bhaduri SB. Fabrication aspects of PLA-CaP/PLGA-CaP composites for orthopedic applications: a review. Acta Biomater 2012;8:1999–2016. 22342596 10.1016/j.actbio.2012.01.031

[12] Mi FL, Lin YM, Wu YB Chitin/PLGA blend microspheres as a biodegradable drug-delivery system: phase-separation, degradation and release behavior. Biomaterials 2002;23:3257–67. 12102197 10.1016/s0142-9612(02)00084-4

[13] Xiong Y, Zeng YS, Zeng CG Synaptic transmission of neural stem cells seeded in 3-dimensional PLGA scaffolds. Biomaterials 2009;30:3711–22. 19375792 10.1016/j.biomaterials.2009.03.046

[14] Gentile P, Chiono V, Carmagnola I An overview of poly(lactic-*co*-glycolic) acid (PLGA)-based biomaterials for bone tissue engineering. Int J Mol Sci 2014;15:3640. 24590126 10.3390/ijms15033640 PMC3975359

[15] Boccaccini AR, Maquet V. Bioresorbable and bioactive polymer/Bioglass® composites with tailored pore structure for tissue engineering applications. Compos Sci Technol 2003;63:2417–29.

[16] Rezwan K, Chen QZ, Blaker JJ Biodegradable and bioactive porous polymer/inorganic composite scaffolds for bone tissue engineering. Biomaterials 2006;27:3413–31. 16504284 10.1016/j.biomaterials.2006.01.039

[17] Jiang LX. Effect of a new surface-grafting method for nano-hydroxyapatite on the dispersion and the mechanical enhancement for poly(lactide-co-glycolide). Express Polym Lett 2013;8:133–41.

[18] Zhao J, Guo LY, Yang XB Preparation of bioactive porous HA/PCL composite scaffolds. Appl Surf Sci 2008;255:2942–6.

[19] Yin Y, Ye F, Cui J Preparation and characterization of macroporous chitosan-gelatin/beta-tricalcium phosphate composite scaffolds for bone tissue engineering. J Biomed Mater Res: Part A 2003;67:844. 10.1002/jbm.a.10153 14613233

[20] Li H, Chang J. Fabrication and characterization of bioactive wollastonite/PHBV composite scaffolds. Biomaterials 2004;25:5473–80. 15142728 10.1016/j.biomaterials.2003.12.052

[21] Chen QZ, Thouas GA. Fabrication and characterization of sol-gel derived 45S5 Bioglass®-ceramic scaffolds. Acta Biomater 2011;7:3616–26. 21689791 10.1016/j.actbio.2011.06.005

[22] Ou J, Kang Y, Huang Z Preparation and in vitro bioactivity of novel merwinite ceramic. Biomed Mater 2008;3:015015. 18458502 10.1088/1748-6041/3/1/015015

[23] Chen X, Zhang M, Pu X Characteristics of heat-treated plasma-sprayed CaO–MgO–SiO_2_ -based bioactive glass–ceramic coatings on Ti–6Al–4V alloy. Surf Coat Technol 2014;249:97–103.

[24] Wu C, Chang J, Ni S In vitro bioactivity of akermanite ceramics. J Biomed Mater Res: Part A 2006;76A:73–80. 10.1002/jbm.a.30496 16224776

[25] Wu C, Jiang C. Degradation, bioactivity, and cytocompatibility of diopside, akermanite, and bredigite ceramics. J Biomed Mater Res: Part B Appl Biomater 2006;83B:153–60. 10.1002/jbm.b.30779 17318828

[26] Zhai W, Lu H, Chen L Silicate bioceramics induce angiogenesis during bone regeneration. Acta Biomater 2012;8:341–9. 21964215 10.1016/j.actbio.2011.09.008

[27] Huang Y, Jin X, Zhang X In vitro and in vivo evaluation of akermanite bioceramics for bone regeneration. Biomaterials 2009;30:5041–8. 19545889 10.1016/j.biomaterials.2009.05.077

[28] Kang Y, Yao Y, Yin G A study on the in vitro degradation properties of poly(L-lactic acid)/beta-tricalcuim phosphate (PLLA/beta-TCP) scaffold under dynamic loading. Med Eng Phys 2009;31:589–94. 19131266 10.1016/j.medengphy.2008.11.014

[29] Zhang M, Chen X, Pu X Different effects of a novel CaO-MgO-SiO_2_-based multiphase glass-ceramic on cell behaviors of normal and cancer cells in vitro. Colloids Surf B Biointerfaces 2014;116:1–8. 24441176 10.1016/j.colsurfb.2013.12.046

[30] Hanuza J, Ptak M, Mączka M Polarized IR and Raman spectra of Ca 2 MgSi 2 O 7, Ca 2 ZnSi 2 O 7 and Sr 2 MgSi 2 O 7 single crystals: temperature-dependent studies of commensurate to incommensurate and incommensurate to normal phase transitions. J Solid State Chem 2012;191:90–101.

[31] Kalinkina EV, Kalinkin AM, Forsling W Sorption of atmospheric carbon dioxide and structural changes of Ca and Mg silicate minerals during grinding: I. Diopside. Int J Mineral Process 2001;61:273–88.

[32] Liang JZ. Reinforcement and quantitative description of inorganic particulate-filled polymer composites. Compos Part B Eng 2013;51:224–32.

[33] Yang F, Cui W, Xiong Z Poly(L,L-lactide-co-lycolide)/tricalcium phosphate composite scaffold and its various changes during degradation in vitro. Polym Degrad Stab 2006;91:3065–73.

[34] Li H, Chang J. pH-compensation effect of bioactive inorganic fillers on the degradation of PLGA. Compos Sci Technol 2005;65:2226–32.

[35] Filipowska J, Pawlik J, Cholewa-Kowalska K Incorporation of sol–gel bioactive glass into PLGA improves mechanical properties and bioactivity of composite scaffolds and results in their osteoinductive properties. Biomed Mater 2014;9:065001. 25329328 10.1088/1748-6041/9/6/065001

[36] Jones JR. Review of bioactive glass: from Hench to hybrids. Acta Biomater 2013;9:4457–86. 22922331 10.1016/j.actbio.2012.08.023

[37] Hench LL, Polak JM. Third-generation biomedical materials. Science 2002;295:1014. 11834817 10.1126/science.1067404

[38] Hallab NJ, Bundy KJ, O'Connor K Cell adhesion to biomaterials: correlations between surface charge, surface roughness, adsorbed protein, and cell morphology. J Long Term Eff Med Implants 1995;5:209–31. 10172729

[39] Valerio MP P, Goes AM, Leite MF. The effect of ionic products from bioactive glass dissolution on osteoblast proliferation and collagen production. Biomaterials 2004;25:2941–8. 14967526 10.1016/j.biomaterials.2003.09.086

[40] Xynos ID, Edgar AJ, Buttery LDK Ionic products of bioactive glass dissolution increase proliferation of human osteoblasts and induce insulin-like growth factor II mRNA expression and protein synthesis ⋆. Biochem Biophys Res Commun 2000;276:461–5. 11027497 10.1006/bbrc.2000.3503

[41] Marom R, Shur I, Solomon R Characterization of adhesion and differentiation markers of osteogenic marrow stromal cells. J Cell Physiol 2005;202:41–8. 15389528 10.1002/jcp.20109

[42] Mizuno M, Fujisawa R, Kuboki Y. The effect of carboxyl-terminal propeptide of type I collagen (c-Propeptide) on collagen synthesis of preosteoblasts and osteoblasts. Calcif Tissue Int 2000;67:391–9. 11136538 10.1007/s002230001150

[43] Franceschi RT. The developmental control of osteoblast-specific gene expression: role of specific transcription factors and the extracellular matrix environment. Crit Rev Oral Biol Med 1999;10:40–57. 10759426 10.1177/10454411990100010201

[44] Gough JE, Notingher I, Hench LL. Osteoblast attachment and mineralized nodule formation on rough and smooth 45S5 bioactive glass monoliths. J Biomed Mater Res Part A 2004;68A:640–50. 10.1002/jbm.a.20075 14986319

[45] Fei L, Wang C, Xue Y Osteogenic differentiation of osteoblasts induced by calcium silicate and calcium silicate/β-tricalcium phosphate composite bioceramics. J Biomed Mater Res: Part A 2012;100B:1237–44. 10.1002/jbm.b.32688 22454365

